# Rooting and Dating Large SARS-CoV-2 Trees by Modeling Evolutionary Rate as a Function of Time

**DOI:** 10.3390/v15030684

**Published:** 2023-03-05

**Authors:** Xuhua Xia

**Affiliations:** 1 Department of Biology, University of Ottawa, Marie-Curie Private, Ottawa, ON K1N 9A7, Canada; xxia@uottawa.ca; Tel.: +1-613-562-5718; 2 Ottawa Institute of Systems Biology, University of Ottawa, Ottawa, ON K1H 8M5, Canada

**Keywords:** tip-rooting, SARS-CoV-2, viral evolution, evolutionary rate, TRAD, tip-dating, MRCA, COVID-19

## Abstract

Almost all published rooting and dating studies on SARS-CoV-2 assumed that (1) evolutionary rate does not change over time although different lineages can have different evolutionary rates (uncorrelated relaxed clock), and (2) a zoonotic transmission occurred in Wuhan and the culprit was immediately captured, so that only the SARS-CoV-2 genomes obtained in 2019 and the first few months of 2020 (resulting from the first wave of the global expansion from Wuhan) are sufficient for dating the common ancestor. Empirical data contradict the first assumption. The second assumption is not warranted because mounting evidence suggests the presence of early SARS-CoV-2 lineages cocirculating with the Wuhan strains. Large trees with SARS-CoV-2 genomes beyond the first few months are needed to increase the likelihood of finding SARS-CoV-2 lineages that might have originated at the same time as (or even before) those early Wuhan strains. I extended a previously published rapid rooting method to model evolutionary rate as a linear function instead of a constant. This substantially improves the dating of the common ancestor of sampled SARS-CoV-2 genomes. Based on two large trees with 83,688 and 970,777 high-quality and full-length SARS-CoV-2 genomes that contain complete sample collection dates, the common ancestor was dated to 12 June 2019 and 7 July 2019 with the two trees, respectively. The two data sets would give dramatically different or even absurd estimates if the rate was treated as a constant. The large trees were also crucial for overcoming the high rate-heterogeneity among different viral lineages. The improved method was implemented in the software TRAD.

## 1. Introduction

Any viral outbreak would raise the question of when and where a zoonotic transmission event (or a lab-leak event) occurred. The “When” question is answered approximately by the estimation of two parameters, the evolutionary rate (*µ*) and the date of the most recent common ancestor (*T_A_*) of representative viral strains [1,2,3,4,5]. Modern sequencing technology can quickly generate many viral genomic sequences from which a viral tree can be constructed. If the viral tree can be properly rooted, then the parameters *µ* and *T_A_* can be estimated given a tree of viral strains with sample collection times used for calibrating a molecular clock [6,7,8,9,10,11]. The most recent common ancestor of SARS-CoV-2 was dated in two recent studies to October–November 2019 [12] and mid-August [5], respectively. Using viral genomes from China, Pekar et al. [13] inferred the first cryptic infection of SARS-CoV-2 to span the interval between mid-October and mid-November, 2019.

The estimated evolutionary rate based on uncorrelated relaxed clock models varies from low values such as 0.0006 [14] and 0.000605 [15] to substantially higher values of 0.001793 [16] and 0.0024 [17]. Intermediate rates have also been obtained in a number of studies including strict clock [5,18] and uncorrelated relaxed clock [18,19].

These dating studies share two problems. Firstly, although uncorrelated relaxed clock models allow different lineages to have different evolutionary rates (i.e., *μ* values), they do not model *μ* as a function of time. However, *μ* may experience acceleration during the origin of various dominant variants [20]. Severe bias in the estimation of *μ* could result when *μ* increases or decreases with time [5]. Secondly, these dating studies, including the most recent Pekar et al. [21] but with the exception of Xia [5], only used SARS-CoV-2 genomes sampled in 2019 and early 2020. The justification of only including early SARS-CoV-2 genomes is the assumption that a zoonotic transmission occurred in Wuhan and that the culprit was immediately captured [16,21], so that only the SARS-CoV-2 genomes obtained in 2019 and first few months of 2020 (resulting from the first wave of global expansion from Wuhan) are needed for dating. All subsequent genomes have descended from these early strains and consequently share the same root. However, it is possible that early SARS-CoV-2 strains may have circulated in places other than Wuhan at the same time or even earlier than the Wuhan strains. Including more SARS-CoV-2 genomes would increase the chance that the descendants of such non-Wuhan strains are included in dating and rooting the SARS-CoV-2 tree.

I will present an improved method for rooting large viral trees and dating the common ancestor by modeling the evolutionary rate as a function of time. I applied the method to the rooting and dating of two large SARS-CoV-2 trees released by NCBI on 3 April 2021 and 7 May 2022, respectively, with 83,688 and 970,777 full-length high-quality SARS-CoV-2 genomes that contain full sample collection dates. *T_A_* was dated highly concordantly to 2019-06-12 and 2019-07-07 with the two trees, respectively. Treating the evolutionary rate as a constant would generate highly divergent and unreasonable estimates from these two trees.

## 2. An Improved TRAD Method

I will follow the previous convention of referring to the tip-rooting and ancestor-dating method as the TRAD method [5,22] and detail the rationale of its improvement. The TRAD method takes two steps to date the viral tree. The first step is to estimate the root (the rooting step) which is time-consuming. For an unrooted viral tree without an appropriate outgroup to root the tree, the root could be anywhere, so the root is taken as a parameter and estimated. Once the root is estimated, the root-to-tip distance can be computed. The second step is the dating step by using the root-to-tip distance and the viral collection time. The improvement detailed in this manuscript has been implemented in software TRAD [22] freely available at http://dambe.bio.uottawa.ca/TRAD/TRAD.aspx (accessed on 7 May 2022).

### 2.1. The Rooting Step

**Figure 1:**
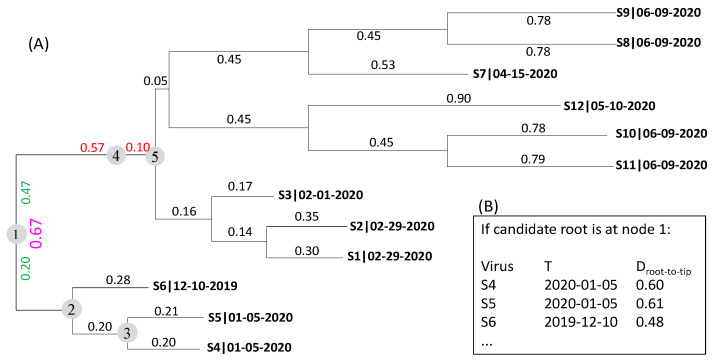
Conceptual illustration of the statistical framework for rooting the tree and dating the most recent common ancestor of the sampled genomes. (**A**) An unrooted viral tree with viral names in the format of Name|T where T is the collection date (mm-dd-year). Branch lengths are shown next to individual branches. Five internal nodes are numbered 1, 2, 3, 4, and 5, respectively. The branch length (0.67) between nodes 2 and 5 is bisected into the two green numbers by internal node 1, and into two red numbers by internal node 4. The root is unknown and could be anywhere along the branches. (**B**) The root-to-tip distance (*D*) when the root is placed at internal node 1.

The rooting step is based on the relationship between two quantities, the root-to-tip distance (*D*) and the sample collection time (*T*), given a phylogenetic tree (Figure 1A). The sequence name in the tree is made of two parts, sequence name and sampling time, separated by a vertical bar (Figure 1A). Branch lengths are indicated by numbers next to the branches. The tree in Figure 1A is unrooted but drawn as if it is rooted. The branch length between internal nodes 2 and 5 (Figure 1A) is 0.67. If the root is placed at internal node 1 (Figure 1A), then the branch length is bisected into two branch segments 0.20 and 0.47 colored in green (Figure 1A). If the root is placed at internal node 4, then the branch length is bisected into two segments 0.57 and 0.10 colored in red (Figure 1A). The root could be anywhere along the branches. Internal nodes 1 to 5 (Figure 1A) are a few candidate rooting positions. The rooting step will place the root at all positions along the tree to find the best root. We need to develop a criterion to choose the best root.

From each candidate root, one can obtain a set of *D* values between the candidate root and each tip. If the rooting point happens to be the true root, then *D* is expected to have the highest correlation to *T* assuming a molecular clock. *T* does not change with rooting positions, but *D* does. For example, if the root is at the branch indicated by the internal node 2, then *D* is 0.28 for S6, and 0.40 and 0.41, respectively, for S4 and S5. If the candidate root is at internal node 3, then *D* is 0.20 and 0.21, respectively, for S4 and S5, and 0.48 for S6. Figure 1B shows several *D* values if the internal node 1 is taken as the root.

Table 1 shows the different *D* values when the root is placed at internal nodes 1, 2, 3 and 4, respectively, where D_i_ is the root-to-tip distance when the root is placed at internal node *i* in Figure 1A. The first two columns do not change, but the last four columns differ with where the root is placed. Different computer programs use different date as day 1. The R package uses 1 January 1970 (1970-01-01) as day 1 but EXCEL uses 1900-01-01 as day 1. I will take the EXCEL convention of using 1900-01-01 as day 1, so 2019-12-10 is 43,809, 2020-01-05 is 43,835, and so on. Using different dates as day 1 does not affect rooting and dating.

**Figure 2:**
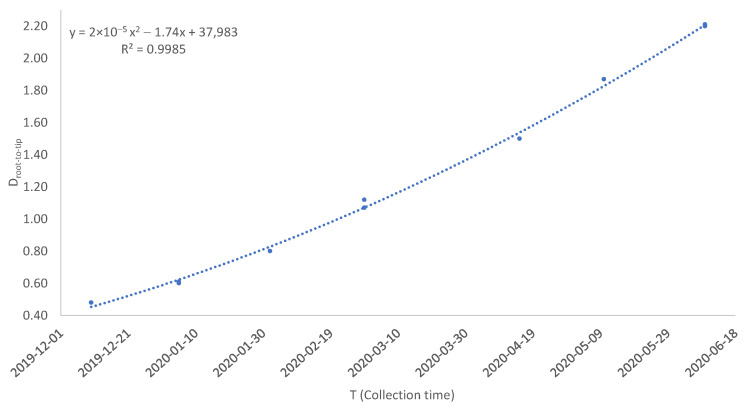
The root-to-tip distance (*D*) increases with viral sample collection time (*T*). Dot color codes are blue for *D*_1_, orange for *D*_2_, black for *D*_3_ and red for *D*_4_. The order-2 polynomial regression line was fitted for *D*_1_ and *T* only.

The rooting step depends on the relationship between *D* and *T*. *D*_1_ (Table 1) has the strongest relationship to *T* relative to *D*_2_, *D*_3_, and *D*_4_ (Figure 2). In previous implementations of the rooting method [5,8,22], the evolutionary rate *µ* is assumed to be constant so the relationship between *D* and *T* is linear: $D=\mu \left(T-T_{A}\right)=-\mu T_{A}+\mu T$. Thus, the rooting point that yields the strongest linear relationship (e.g., the highest Pearson correlation) between *D* and *T* is taken as the estimated root. This method has been implemented in TempEst [8], DAMBE [23] and TRAD [5,22]. For example, the Pearson correlation between *T* and the four candidate rooting positions (Table 1) shows that internal node 1 (Figure 1A) is a better rooting position than internal nodes 2, 3 and 4. However, the internal node 1 is not the best root with *r* as the criterion. Shifting the internal node 1 towards internal node 2 by 0.08709 will achieve the highest *r* of 0.9962 between *T* and the root-to-tip distance designated *D_max.r_* (Table 1). Moving the root to any other point along the branches will reduce *r*. This approach with a constant *µ* and consequently a linear relationship between *D* and *T* will be referred to as Model 1.

One can bootstrap or jackknife $D_{i}$ and $T_{i}$ values to obtain a series of bootstrap/jackknife samples and a corresponding series of $\mu_{j}$ values, one from each bootstrap/jackknife sample. This resampling method of estimating the variation of μ has been implemented in a previous version of TRAD [5]. An alternative approach of estimating the variation of μ is to obtain $\mu_{i}=D_{i}/(T_{i}-T_{A})$ for each viral genome *i*, and then resample these resulting $\mu_{i}$ values. The final *µ* is simply characterized by the mean and variance of these individual $\mu_{i}$ values.

A shortcoming of the method above is that μ is not allowed to change over time. Similarly, an uncorrelated relaxed clock used in viral dating also does not model μ as a function of time, although different lineages can have their own $\mu_{i}$ distributions. While autocorrelated relaxed clock [24,25] would accommodate the change of evolutionary rate over time, such a clock is rarely used in the tip-dating of viral trees and its performance is uncertain.

The problem with Model 1 above is that *µ* is often not constant over time, as I will show empirically later. This is also illustrated with the tree in Figure 1. The points of *D* over *T* do not fall on a straight line, but exhibit a trend with *D* increasing with *T* at an increasing rate (Figure 2). The simplest extension of a constant *µ* is a linear function of *µ* over *t*, i.e., μ=a+bt. This implies *D* as

(1) $$D=\int_{T_{A}}^{T}\left(a+bt\right)dt=-\left(aT_{A}+\frac{b}{2}T_{A}^{2}\right)+aT+\frac{b}{2}T^{2}=B_{0}+B_{1}T+B_{2}T^{2}$$

which is an oder-2 polynomial from which the parameters can be estimated by order-2 polynomial regression. Obviously, $T=T_{A}$ when D=0. This order-2 polynomial function in Equation (1) will be referred to as Model 2 in contrast to the previous Model 1 of $D=B_{0}+B_{1}T$. One may still criticize Model 2 as insufficient for approximating the biological reality. However, it does represent an incremental improvement over a constant *µ* without requiring too much additional computation time.

Equation (1) and Figure 2 suggest that the coefficient of determination (*R*^2^) based on Equation (1) is more appropriate than *r* as a criterion to choose the best rooting position. With a dependent variable *y* and two *x* variables *x*_1_ and *x*_2_, *R*^2^ is

(2) $$R^{2}=\frac{r_{x_{1}y}^{2}+r_{x_{2}y}^{2}-2r_{x_{1}y}r_{x_{2}y}r_{12}}{1-r_{12}^{2}}$$

*R*^2^ values have also been shown in the last row in Table 1 for the four candidate roots as well as for the root with the maximum *r*. With *R*^2^ as the criterion, the optimal root is arrived at by shifting Node 1 towards Node 4 by a distance of 0.006638 (Figure 1). This would yield the maximum *R*^2^ = 0.99854.

Model 1 and Model 2 can be evaluated with a likelihood rate test. For data in Table 1, the log-likelihood (lnL) is 19.381 for Model 1 and 26.218 for Model 2. The likelihood ratio chi-square is 13.674. With one degree of freedom, *p* = 0.0002, so we reject Model 1 and prefer Model 2.

The rooting step is time-consuming with a large tree containing nearly a million genomes because the algorithm needs to traverse all branches along the tree and compute millions of *R*^2^ values to find the optimal rooting position along the tree with the maximum *R*^2^. TRAD [5,22] is currently the only software package that can root a tree with so many leaves. It took less than a day to root the Apr3_21 tree on a regular desktop computer with an 11th Gen Intel(R) Core(TM) i7-11700 and 64 GB of memory, but a week to root the May7_22 tree.

### 2.2. The Dating Step

I will contrast the two models in estimating *T_A_*, one assuming *µ* as a constant and the other modelling *μ* as a linear function of time. We have two variables, *D* and *T* illustrated in the previous section, to estimate *T_A_* (the time of origin of the common ancestor of the sampled SARS-CoV-2 genomes). In the dating step, *D* is the root-to-tip distance from the best root. When *µ* is constant, *D* and *T* are linearly related as follows:

(3) $$D=\mu \left(T-T_{A}\right)=-\mu T_{A}+\mu T$$

Therefore, regressing *D* over *T* will give us $-\mu T_{A}$ as the intercept and *µ* as the slope. For example, regressing *D_max.r_* in Table 1 on *T* yields $\mu =1.08986\times 10^{-2}$ (changes/day/genome) and an intercept of −477.16399. Setting $-\mu T_{A}=477.16399$, we get *T_A_* = 43,782.26 which is equivalent to 13 November 2019. From Equation (3), it is also clear that $T_{A}=T$ when *D =* 0. This approach has been applied to the estimation of $T_{A}$ of sampled SARS-CoV-2 genomes in a previous study by assuming a constant *µ* [5]. However, this approach would be problematic when *µ* is not constant as shown in Figure 2. Not only will it bias the estimate of *T_A_* towards a more recent date, but also generate inconsistent estimates of $T_{A}$ depending on which period the viral genomes were sampled. For example, if we separate the data into two groups, with group 1 including the data collected before 2020-03-10 and group 2 including the data after 2020-03-10, then group 2 will date *T_A_* to a more recent time than group 1 because *µ* increases over time in Figure 2.

For Model 2, the parameter *T_A_* can be estimated as before by setting *D* = 0 in Equation (1), and solving the resulting quadratic equation. The two roots of the function are

(4) $$T_{A}=\frac{-B_{1}\pm \sqrt{B_{1}^{2}-4B_{0}B_{2}}}{2B_{2}}$$

For real SARS-CoV-2 data, the discriminant is always very close to 0, so $T_{A}\approx -B_{1}/(2B_{2})$.

When we take $D_{max.R^{2}}$ in Table 1 as *D*, the regression of *D* over *T* and *T*^2^ gives us $B_{0}=39,848.83964,$ $B_{1}=-1.82494,B_{2}=2.08940\times 10^{-5}$. Therefore, $\mu \left(T\right)=-1.82494+4.17879\times 10^{-5}T$. Our previous treatment of *µ* as a constant yields $\mu =1.08986\times 10^{-2}$, which would be the evolutionary rate on 11 April 2020 given μ(T). The discriminant turned out to be −0.0000055554 and may be taken as 0, so $T_{A}\approx -\frac{B_{1}}{2B_{2}}=43,671.4$ which is 2019-07-25. This is 110 days earlier than the estimate of $T_{A}$ when *µ* is taken as a constant. The variance of $T_{A}$ can be estimated by bootstrapping.

## 3. Results

The rooting and dating results are based on two large SARS-CoV-2 trees released by NCBI at https://www.ncbi.nlm.nih.gov/labs/virus/vssi/#/precomptree (accessed on 7 May 2022). These trees are based on full-length and high-quality SARS-CoV-2 genomes built with the phylogenetic infrastructure for characterizing viral variation and viral phylogenies [26]. One tree was downloaded on 3 April 2021, with 86,582 SARS-CoV-2 genomes, and the other on 7 May 2022, with 1,274,974 SARS-CoV-2 genomes. These two trees will be referred to as the Apr3_21 tree and the May7_22 tree, respectively. After removing genomes without complete date information (e.g., only year or only year and month but no date), 83,688 leaves remain in the Apr3_21 tree and 970,777 leaves remain in the May7_22 tree. They are available at http://dambe.bio.uottawa.ca/Trees_Apr3_21_and_May7_22.zip (accessed on 7 May 2022) which contains the two original trees (Apr3_21.dnd and May7_22.dnd) as well as the results after rooting and dating these two trees (Apr3_21_rooted_dated.txt and May7_22_Rooted_Dated.txt).

**Figure 3:**
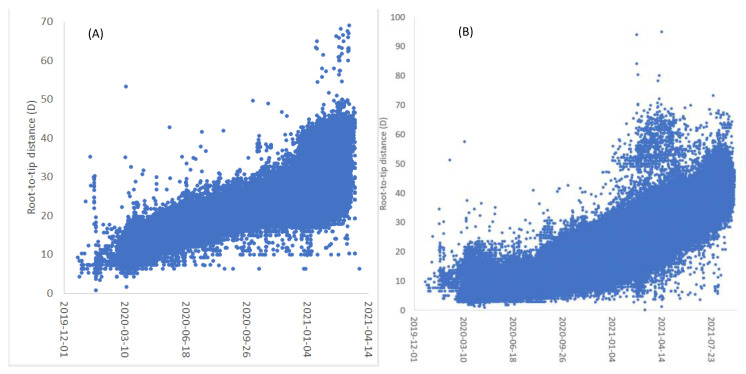
Changes in evolutionary rate over time visualized by plotting root-to-tip distance (*D*) over viral sample collection time (horizontal axis). *D* is from the optimal root estimated as described. (**A**) Relationship between *D* and *T* from the tree released by NCBI on 3 April 2021, and (**B**) on 7 May 2022.

The relationship between the root-to-tip distance (*D*) from the best root and the viral sample collection time (*T*), are shown for both the Apr3_21 tree (Figure 3A) and the May7_22 tree (Figure 3B). The evolutionary rate is roughly linear for the Apr3_21 tree in Figure 3A, but changes a lot for the May7_22 tree in Figure 3B. This means that Model 1 and Model 2 will have similar estimates of *T_A_* given the Apr3_21 tree, but differ a lot in the estimated *T_A_* for the May7_22 tree.

The dating results from the two trees are summarized in Table 2. As expected, *T_A_* estimated from Model 1 and Model 2 are similar with the Apr3_21 tree (*T_A_* is 2019-08-16 from Model 1 and 2019-06-12 for Model 2, Table 2). However, for the May7_22 tree (Figure 3B) where *μ* is apparently not constant, Model 1 generated an absurd estimate of *T_A_* of 4 March 2020 (Table 2). In contrast, both the Apr3_21 tree and the May7_22 tree under Model 2 generated consistent estimates of *T_A_* (2019-06-12 and 2019-07-07, respectively, Table 2).

The results in Table 2 allow one to use likelihood ratio tests or information-theoretic indices such as AIC and BIC to evaluate the two models. For the Apr3_21 tree, the likelihood ratio chi-square is 272.558. With one degree of freedom, p = 3.14 × 10^−61^, which led to a strong rejection of Model 1 in favor of Model 2. For the May7_22 tree, the p value is even smaller, as one would have expected by contrasting Figure 3A and Figure 3B where the relationship between *D* and *T* visually curves more in Figure 3B than in Figure 3A. The results of the likelihood ratio tests are consistent with AIC and BIC as model-selection criteria. Both AIC and BIC strongly favor Model 2 against Model 1 (Table 2).

One might argue that *μ* may change with time in a manner more complicated than a linear function of time can sufficiently describe. I may defend Model 2 with two lines of arguments. First, Model 2 accounts for 74.55% and 84.36% of the total variance in *D* (Table 2), representing an excellent fit to the empirical data. Second, treating *μ* as a linear function of time represent at least an incremental improvement over the convention of treating *μ* as a constant.

## 4. Discussion

The TRAD method developed and used in this paper has advantages and disadvantages. The most obvious statistical problem is caused by coancestry, so that the $D_{i}$ values are not independent of each other. Different $D_{i}$ values could traverse the same internal branch. For this reason, TRAD uses resampling methods for estimating the parameter variance instead of deriving the parameter variance directly from the regression. Related to this coancestry problem is that an unusual internal branch could exert its effect of parameter estimation through all $D_{i}$ values that include this branch. The effect of such an unusual internal branch would be particularly pronounced if the total sample size is small. In this context, the TRAD method is advantageous because it can root and date very large trees that are impossible with other methods. Large samples are generally more robust against one or a few unusual internal branches. Below, I examine more critically the pros and cons of using large trees, as well the assumptions of the molecular clock.

### 4.1. The Pros and Cons of Using Large Trees

Suppose we sample 500 SARS-CoV-2 genomes globally over a three-month period from the first Wuhan outbreak (say by 31 March 2020), and if these 500 genomes had evolved at a constant rate with little rate heterogeneity. These 500 genomes would allow us to reach a good estimate of *T_A_*. If a new strain originated at the end of March 2020 from one of those early lineages, displaced the original strains, and evolved at a rate different from the original, then including the genomes of these new strains would likely introduce a bias in the estimation of *T_A_*. In this scenario, it is better to have a small tree of the 500 genomes than a large tree including new strains that may have a very different rate of evolution.

Previous dating and rooting studies used small trees partly because there has been no software other than TRAD that can perform rooting and dating with trees containing a million or more leaves and partly (and perhaps more importantly) because of the following assumption. That is, a zoonotic transmission occurred in Wuhan and the new pathogen was immediately caught [16,21]. Therefore, all subsequent SARS-CoV-2 genomes would share the same root as the early ones and do not need to be included for rooting and dating the common ancestor. Including all later viral genomes with potentially drastically different evolutionary rates may actually make the estimate of *T_A_* less reliable.

It is possible that the evolutionary rate has changed soon after the zoonotic transmission, e.g., increasing as specified by a linear function. In this case, one should use the quadratic model as specified in Equation (1). However, it is also possible that the rate of evolution changes in a complicated way beyond what could be accommodated by Equation (1). In this latter case, the advantage of Equation (1) is not clear, although one may argue that the approximation of a dynamic relationship by a linear function is probably better than approximation by a constant.

Large trees do have two other advantages. First, it is possible that SARS-CoV-2 might have cryptically circulated for some time in places other than Wuhan. In this case, if we include only the SARS-CoV-2 genomes from Wuhan and those that can be traced to Wuhan, then the root of the viral tree will necessarily be traced to Wuhan [5,27]. By using large trees with a million SARS-CoV-2 genomes, we have an increased chance of sampling those descendants of the putative cryptically circulating non-Wuhan strains.

**Figure 4:**
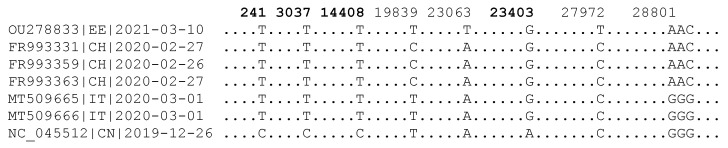
Sequence alignment summarizing all genomic variation among seven SARS-CoV-2 genomes. The genome sequence name is in the form of ACCN|Country_code|Collection_date. EE: Estonia; CH: Switzerland; IT: Italy; CN: China. The four nucleotide sites characterizing the D614G lineage are in bold.

The second advantage of using large trees is to overcome the high rate-heterogeneity among different SARS-CoV-2 lineages [5]. It might help to illustrate this with concrete genomes. The sequence alignment in Figure 4 highlights the slow evolution of a SARS-CoV-2 genome from Estonia (OU278833|EE|2021-03-10). The three viral genomes from Switzerland (CH) were sampled more than a year ago, but there are only three nucleotide differences between the Estonia-derived sequence and the Switzerland-derived sequences. In particular, the nucleotide T at site 19,839 of OU278833 represents an ancestral state in comparison with the reference genome from China (Figure 4), so that the nucleotide difference between OU278833 and the Switzerland SARS-CoV-2 sequences should be attributed to mutation in the latter rather than in the former. Therefore, only two nucleotide changes, one at site 23,063 and the other at site 27,972, represent changes along the OU278833 lineage from a parsimoniously reconstructed common ancestor of the first four SARS-CoV-2 sequences in Figure 4. During the same period, many other genomic sequences have accumulated 20 or 30 nucleotide differences. There are many such examples in SARS-CoV-2 genomic data. This rate heterogeneity is also obvious in Figure 3. A small sample is highly likely to generate a biased estimate of *T_A_*.

### 4.2. Strict and Uncorrelated Relaxed Clock

**Figure 5:**
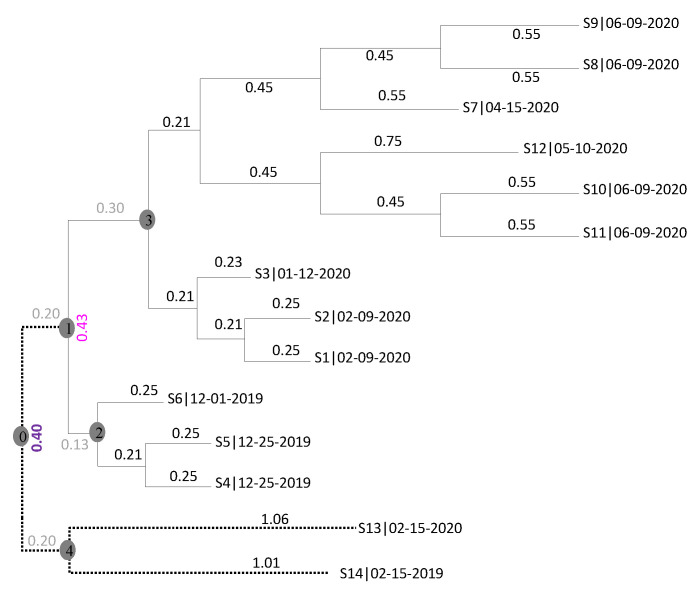
A fictitious viral tree with 14 viral genomes (S1 to S14) taken at different times shown as part of OTU names. Branch lengths are indicated next to the branch. An unrooted tree including S1 to S12 but excluding S13 and S14 would have only one branch connecting Node 2 and Node 3, with the branch length indicated by the pink 0.43. Similarly, an unrooted tree of all 14 samples has only one branch connecting Node 1 and Node 4, with the branch length indicated by the purple 0.40. Numbered circles are nodes mentioned in the text.

Both a strict clock and an uncorrelated relaxed clock have been used in rooting and dating the common ancestor of SARS-CoV-2 genomes. I wish to highlight a conceptual problem that could potentially bias dating with either of the two clock models. Figure 5 shows a fictitious viral tree. One ancestral node, indicated by Node 1 (circled and shaded in gray), is highly successful in leaving many descending lineages represented by S1–S12. The other ancestral node, indicated by Node 4, is less successful with only two descending lineages sampled (S13 and S14). Without S13 and S14 and the dotted branches, any dating method would find the root indicated by Node 1, dated to 2019-10-14.

Including S13 and S14 would render this root at Node 1 unplausible given a strict clock, because the root-to-tip distance for S13 and S14 would be too long if the root is at Node 1. With a strict clock, we would find a root close to Node 0 (circled and shaded in gray), dated to mid-September 2019. However, with an uncorrelated relaxed clock (which has many different versions), the dating method would happily keep the common ancestor of the 14 viral lineages at Node 1, by allowing the evolutionary rate leading to S13 and S14 to be greater than that for the other 12 lineages (from S1 to S12).

Now is the root at Node 1 or Node 0? With a strict clock, Node 0 is a more plausible root than Node 1. However, with a relaxed clock, especially when one allows dashed branches to have more freedom in the evolutionary rate, Node 1 can be just as plausible (and could indeed be made even more plausible by adding some lineages with certain branch lengths and collection times). In one of the early studies [19], when the among-lineage rate variation is constrained, the strict clock fits data better than the uncorrelated lognormal clock. However, when such a constraint is relaxed, the uncorrelated lognormal clock fits the data just as well as the strict clock or even better.

One often cannot discriminate between these two rooting hypotheses (Node 1 or Node 0) without additional information. Node 1 might indeed be the true root and the dotted branches in Figure 5 represent an accelerated evolutionary rate. It might also be true that the evolution indeed follows a strict clock, that the true root is at Node 0, and that the uncorrelated relaxed clock biased the estimate and misidentified the true root as Node 1. Some researchers therefore resorted to additional information. Rambaut et al. [16], Pekar et al. [21] and Worobey et al. [28] favored the hypothesis that the zoonotic event occurred in Huanan Seaford Market in Wuhan and that the culprit was immediately caught. If S1 to S12 were all from Wuhan, but S13 and S14 were from elsewhere, then dating does not need to include S13 and S14. One only needs to include the early viral lineages in Wuhan and those that can be traced to Wuhan. Similarly, Pekar et al. (2022) limited the SARS-CoV-2 sequences collected by 14 February 2020. If I apply the same approach, then S13 and S14 in Figure 5 would be excluded from the analysis.

The problem above is well known and indeed has often been discussed informally among researchers engaged in developing dating methods. However, researchers who applied the dating method often forgot this potential problem. I should also add that, while Figure 5 is a distance-based illustration, one can have the same illustration with aligned sequences and site-oriented dating methods.

## 5. Conclusions

In summary, the original TRAD method assuming a constant evolutionary rate *µ* is problematic in three ways. First, the assumption is clearly false. Second, it generates widely different estimates of two key parameters, i.e., *µ* and *T_A_* with two different viral phylogenies. Third, when *µ* increases with time, the estimated *T_A_* may be biologically absurd. In contrast, modelling *µ* as a linear function of time instead of a constant eliminates all these problems. I applied this approach to analyzing two large trees released by NCBI on 3 April 2021, and 7 May 2022, including 83,688 and 970,777 high-quality and full-length SARS-CoV-2 genomes, respectively, with complete sample collection dates for the included viral genomes. The most recent common ancestor of the sampled SARS-CoV-2 genomes was dated to 12 June 2019 with the Apr3_21 tree, and 7 July 2019 with the May7_22 tree with 970,777 leaves. The results also highlight the importance of having very large trees because of substantial rate heterogeneity among different SARS-CoV-2 lineages.

## Figures and Tables

**Table 1:** Different root-to-tip distances (*D*_1_, *D*_2_, *D*_3_, and *D*_4_) when the candidate root is placed at internal nodes 1, 2, 3 and 4 in Figure 1A, respectively. *T* is virus collection time in the format of yyyy-mm-dd. The last two rows show (1) the Pearson correlation (r) between *T* and *D* and (2) coefficient of determination (*R*^2^) based on $D=b_{0}+b_{1}T+b_{2}T^{2}$.

Virus	*T*	*D*_1_	*D*_2_	*D*_3_	*D*_4_	*D_max.r_*	$D_{max.R^{2}}$
S4	2020-01-05	0.60	0.40	0.20	0.97	0.51291	0.60664
S5	2020-01-05	0.61	0.41	0.21	0.98	0.52291	0.61664
S6	2019-12-10	0.48	0.28	0.48	0.85	0.39291	0.48664
S1	2020-02-29	1.07	1.27	1.47	0.70	1.15709	1.06336
S2	2020-02-29	1.12	1.32	1.52	0.75	1.20709	1.11336
S3	2020-02-01	0.80	1.00	1.20	0.43	0.88709	0.79336
S7	2020-04-15	1.50	1.70	1.90	1.13	1.58709	1.49336
S8	2020-06-09	2.20	2.40	2.60	1.83	2.28709	2.19336
S9	2020-06-09	2.20	2.40	2.60	1.83	2.28709	2.19336
S10	2020-06-09	2.20	2.40	2.60	1.83	2.28709	2.19336
S11	2020-06-09	2.21	2.41	2.61	1.84	2.29709	2.20336
S12	2020-05-10	1.87	2.07	2.27	1.50	1.95709	1.86336
*r*		0.9953	0.9949	0.9749	0.8580	0.99786	0.99486
*R* ^2^		0.9985	0.9909	0.9538	0.9362	0.99662	0.99854

**Table 2:** Summary of the dating results for the Apr3_21 tree (with 83,688 leaves) and the May7_22 tree (with 970,777 leaves). Note the absurd estimation of *T_A_* under Model 1 with the May7_22 tree.

	The Apr3_21 Tree		The May7_22 Tree	
	Model 1 ^(1)^	Model 2 ^(2)^	Model 1 ^(1)^	Model2 ^(2)^
B_0_^(3)^	−2415.05517	38,679.17639	−3247.86320	122,456.46937
B_1_^(3)^	0.05527	−1.80887	0.073993	−5.61023
B_2_^(3)^	N/A	2.114018 × 10^−5^	N/A	6.425812 × 10^−5^
*µ* ^(4)^	0.05527	$B_{1}+2B_{2}T$	0.073993	$B_{1}+2B_{2}T$
T_A_^(5)^	2019-08-16	2019-06-12	2020-03-04	2019-07-07
*R* ^2 (6)^	0.74468	0.74550	0.82732	0.84360
lnL ^(7)^	−235,496.173	−235,359.894	−2,677,701.465	−2,631,291.332
AIC ^(7)^	470,996.346	470,725.788	5,355,408.930	5,262,588.663
BIC ^(7)^	471,015.015	470,753.793	5,355,444.288	5,262,624.021

^(1)^ Model 1 treats evolutionary rate *µ* as constant. ^(2)^ Model 2 treats evolutionary rate *µ* as a linear function of time, as in Equation (1). ^(3)^ B_0_, B_1_, B_2_: regression coefficients as in Equation (1). ^(4)^ µ: evolutionary rate in number of mutations per genome per day. ^(5)^ T_A_: date of the most recent common ancestor (MRCA) of the sampled genomes. ^(6)^ *R*^2^: coefficient of determination from the order-2 polynomial. ^(7)^ Log-likelihood (lnL) of the models and the associated AIC and BIC for model selection.

## Data Availability

Not applicable.
